# *In-silico* identification of anti-cholera phytochemicals from Indian medicinal plants

**DOI:** 10.1371/journal.pone.0342058

**Published:** 2026-02-02

**Authors:** Khalilur Rahman, Yasmin Akter, Md Selim Reza, Md. Al Amin Pappu, Md. Rezaul Karim, Marzia Sultana, Mohammad Tarequl Islam, Munirul Alam, Md Nurul Haque Mollah, Md. Mamun Monir

**Affiliations:** 1 icddr, b, (International Centre for Diarrhoeal Disease Research, Bangladesh), Dhaka, Bangladesh; 2 Bioinformatics Lab (dry), Department of Statistics, University of Rajshahi, Rajshahi, Bangladesh; 3 Tulane Center for Biomedical Informatics and Genomics, Deming Department of Medicine, School of Medicine, Tulane University, New Orleans, Louisiana, United States of America; 4 Department of Biotechnology & Genetic Engineering, Faculty of Biological Science, Islamic University, Kushtia, Bangladesh; Berhampur University, INDIA

## Abstract

Cholera is a severe diarrheal disease caused by ingestion of food or water contaminated with pathogenic *Vibrio cholerae*. Treatment for cholera includes rehydration therapy and antibiotics to avert death and reduce bacterial burden to prevent rapid transmission of the disease. In addition, in Indian subcontinent, there is historical evidence of using plants for treating cholera. This study was designed to investigate the cholera toxin-inhibitory properties of phytochemicals sourced from Indian medicinal plants. For this, three reported genotypes of cholera toxin subunit B (ctxB) associated with 7PET *V. cholerae* O1 El Tor strains were used as targets in molecular docking. Analysis results showed strong binding affinities (≤−7.5 kcal/mol) for 298 out of 7,607 phytochemicals, with minor variations for the ctxB genotype-specific targets. Multiple phytochemicals from the same plants were identified with high binding affinities, *e.g.*, 101 from *Morus alba,* 24 from *Citrus aurantium,* 17 from *Emblica officinalis,* and 16 from *Capsicum annuum*. However, further analyses, including drug-likeness, pharmacokinetics, and toxicity, identified five promising phytochemical candidates, namely, Abyssinone V (*Azadirachta indica*), Diosgenin (*Achyranthes bidentata*), Yamogenin (*Borassus flabellifer*), and two other unnamed phytochemicals (one from *Azadirachta indica* and one from *Morus alba*) for cholera toxin inhibition. Molecular dynamics simulation using YASARA and GROMACS showed structural stability of the ctxB-phytochemical complexes, while exhibiting adaptive rearrangements of ligand within the active binding sites of the proteins. In the simulations, MM-PBSA binding free energies showed a favorable total binding energy for the complexes. Per-residue energy decomposition analysis identified different highly contributing sets of amino acids to the binding energy with variation for both ctxB genotypes and phytochemicals, suggesting bacterial evolutionary changes may affect binding patterns of the drug candidates. This study suggests five inhibitors of cholera toxin with varying genotypes, which may have potential as an alternative medication for cholera.

## 1. Introduction

Cholera remains a major global health challenge, caused by pathogenic *Vibrio cholerae,* particularly in regions with inadequate hygiene and sanitation [1–3]. It often leads to severe dehydration and is characterized by the profuse release of a significant volume of fluid resembling rice-water diarrhea [1,2]. Nearly 3 million cases of cholera are reported annually, with deaths ranging from 21,000–143,000, and a notable upsurge has been observed since 2021 [4–6]. Cholera was associated with the classical biotype of *V. cholerae* O1 until the sixth pandemic, which began in 1817, whereas the ongoing seventh cholera pandemic, starting in the early 1960s, has been linked to *V. cholerae* O1 El Tor biotype [1]. In 1992, *V. cholerae* O139 emerged in India and subsequently in Bangladesh, producing the same cholera enterotoxin [2,7]. The global rise in El Tor cholera cases may be due to enhanced ability to thrive in aquatic environmental reservoirs, reflected by upregulated gene expression involved in biofilm formation, chemotaxis, and nutrient transport, such as iron, peptides, and amino acids [8,9].

The most effective treatment of cholera is oral rehydration saline (ORS) [10]. However, it alone does not eliminate the pathogen or prevent the mode of cholera toxin [11]. Besides, a two-dose regimen of antibiotics, such as tetracycline, fluoroquinolone, erythromycin, and azithromycin, has been used for the treatment of cholera for a long period of time, aiming to decrease the pathogen burden and reduce disease transmission [12,13]. However, a concerning trend of resistance to antibiotics has been driven by irrational use of these antibiotics, along with other selective pressures [14]. Recent studies observed numerous antibiotic-resistant *V. cholerae* O1 strains carrying multiple antibiotic-resistance genes over the years [15–17]. Therefore, researchers have been considering alternative therapeutic approaches to combat cholera, including vaccines, probiotics, peptides, bacteriophages, and phytochemicals [18–23]. Vaccines have emerged as a promising alternative, but their availability remains limited compared to the high demand [5,24–28]. While some other approaches are under investigation, medicinal plant-derived phytochemicals, in particular, hold significant promise as an alternative therapy [29–32]. In the Indian subcontinent, it is a common practice to use various parts of medicinal plants (such as leaves, bark, roots, and fruits) to treat different diseases. A recent effort led to the development of a database called IMPPAT 2.0 (Indian Medicinal Plants, Phytochemistry And Therapeutics) [33], which stores information about Indian medicinal plants and their compounds for numerous therapeutic uses from traditional books, published articles, and other resources, including cholera.

Cholera toxin is the primary virulence factor of *V. cholerae* infection (cholera) and consists of two subunits, A and B [34]. During infection, the B subunit binds to the primary receptor, the monosialoganglioside GM1, located on the surface of host enterocytes, which allows internalization of the A subunit, which elevates intracellular cAMP levels, triggers the excessive secretion of water and electrolytes, leading to profuse diarrhea [35–37]. Recent studies have revealed that cholera toxin subunit B also binds to fucosylated receptors, such as histo-blood group antigen, and may cause infection, even when GM1 synthesis is inhibited [38–42]. Therefore, we investigated a therapeutic approach using phytochemicals to inhibit *ctxB* from binding to host receptors.

In this study, molecular docking, ADMET, and drug-likeness properties were analyzed to identify the potential plant-derived phytochemicals considering ctxB genotypes (*ctxB1*, *ctxB3*, and *ctxB7*) as targets. The stability of complexes between identified phytochemicals and ctxB was validated by molecular dynamics simulations using YASARA and GROMACS. Furthermore, average MM-PBSA binding free energy was calculated to assess binding favorability, and per-residue energy decomposition analysis was conducted to identify and quantify the energetic contribution of individual amino acids involved in ctxB-phytochemical complexes.

## 2. Materials and methods

### 2.1 Data source and description

Cholera toxin subunit B (*ctxB*) serves as a crucial biomarker for understanding the mechanism of *V. cholerae* infection [34]. The toxin gene initiates infection by binding to the specific host receptors [35–42]. In this study, protein sequences of the *ctxB* were collected from NCBI for the 3D structure prediction and subsequently used for molecular docking analysis.

Different diseases have been treated by using the resources from medicinal plants in the Indian subcontinent [43,44]. The IMPPAT (Indian Medicinal Plants, Phytochemistry and Therapeutics) database listed 4,010 Indian medicinal plants based on the information from more than 100 herbal medicine books, published studies, and other resources [33]. The database provides comprehensive information about the plants traditionally suggested for 1,095 therapeutic uses, including cholera. This study utilized information from the database to identify a list of plants that were traditionally used or suggested for cholera treatment.

#### 2.1.1 Target identification.

Thirteen genotypes of *ctxB* have been reported to date through experimental approaches such as conventional PCR, qPCR, and in-silico analysis, with *ctxB1*, *ctxB3*, and *ctxB7* being linked to the ongoing seventh cholera pandemic [45–57]. The *V. cholerae* O1 El Tor strains primarily contained the *ctxB3* genotype before adopting the classical *ctxB1* genotype [48,49]. In addition, the 2010 Haiti cholera outbreak was associated with another mutant variant of cholera toxin, namely *ctxB7,* which was first reported in Odisha, India, in 1999 [58]. According to recent studies, circulating strains in Asia, Africa, and other endemic regions mostly carry the *ctxB7* genotype [45–59]. In Bangladesh, strains with *ctxB1* and *ctxB7* genotypes co-existed up to 2019, after that, only strains with *ctxB7* were reported [16,46,57,60,61]. Considering these changes over time, we used all three genotypes of *ctxB* as drug targets, with the additional aim to understand the possible changes in the drug candidates due to bacterial evolutionary changes often observed.

#### 2.1.2 Collection of phytochemicals as drug agents.

IMPPAT 2.0 database listed a total of 177 medicinal plants as resource for traditional cholera treatment [33]. Among these, we considered 55 widely known medicinal plants, which are largely available locally in Bangladesh. We then searched these plants in the PubChem database [62] to retrieve all related phytochemicals along with the available 3D structures. Phytochemicals found in multiple plants were listed only once under a specific plant. A total of 7,607 unique phytochemicals from different plants were collected and analyzed in this study (S1 Dataset). This study was entirely based on secondary data. No human participants or animals were involved. Verbal permission to conduct this study was obtained from the research administration (RA) of our institute.

### 2.2 Preprocessing of drug targets and agents

The 3D structures of the *ctxB* genotype were predicted from protein sequences retrieved from NCBI using SWISS-MODEL [63]. Quality of the predicted protein structures was assessed by the Ramachandran plot [64], which was further protonated at 7.4 pH using PDB2PQR [65] and H++ [66]. From protein structures, water molecules, attached ligands, and heteroatoms were removed, and then Kollman charges, the required hydrogen atoms were added by using AutoDockTools [67]. Finally, the processed structures were stored in pdbqt format for molecular docking.

The 3D structures of phytochemicals were downloaded in SDF format from the PubChem database [62]. Energy minimization of the collected phytochemicals was conducted using OpenBabel [68], with the customized MMFF94 force field, applying the steepest descent algorithm with a maximum of 5,000 iterations, and the structures were saved in PDB format. Furthermore, Gasteiger charges were added, and the torsion tree, including both rotatable and non-rotatable bonds in the phytochemicals, was defined using AutoDockTools, and the file was stored in pdbqt format.

### 2.3 Screening of phytochemicals by molecular docking analysis

Grid box was generated to cover the whole protein surface with a default spacing value (0.375 Å) by using AutoDockTools [67], and then a config file was manually created for 3D coordinates and size of the box with exhaustiveness value equal to 8. For *ctxB1*, the grid box was centered at coordinates x = −6.433, y = 11.478, and z = 10.41, with a box size of 78 × 66 × 84 grid points along the x, y, and z axes, respectively. Similarly, for *ctxB3*, the center was positioned at x = −11.478, y = −15.787, and z = 15.47, with a grid box size of 80 × 58 × 76 grid points. And, for *ctxB7*, the grid box was centered at x = −7.05, y = 13.034, and z = 9.081, with 78 × 60 × 78 grid points. Molecular docking was performed using AutoDock Vina [69,70], which employs a scoring function based on steric complementarity, hydrophobic interaction, hydrogen bonding, and a penalty for ligand flexibility to estimate binding affinities of the phytochemical and target protein complexes, and uses a gradient-based conformational search to identify the most stable binding pose (lowest energy). Phytochemicals that met the defined threshold of binding affinity were shortlisted for further ADMET analysis.

### 2.4 ADME/T analysis

The ADME/T (Absorption, Distribution, Metabolism, Excretion, and Toxicity) analysis of the top-ranked phytochemicals was conducted to facilitate the prediction of drug-like properties, pharmacokinetics, and pharmaceutical characteristics and toxicity of the phytochemicals by using four different online tools, such as SwissADME [71], pkCSM [72], admetSAR 3.0 [73], and ADMETlab 3.0 [74]. SwissADME, pkCSM, and admetSAR 3.0 were used to evaluate ADME parameters, while pkCSM, admetSAR 3.0, and ADMETlab 3.0 were used to assess the toxicity parameters. A consensus-based approach was applied, where the outcome of a parameter was accepted only if at least two tools agreed. Lipophilicity (XLOGP3), topological polarity surface area (TPSA), solubility (log S), rotatable bonds, and Lipinski’s Rule of Five (Molecular weight ≤ 500 daltons, MLOGP ≤ 4.15, hydrogen bond donors (HBD) ≤ 5, and hydrogen bond acceptors (HBA) ≤ 10) [75] were applied for investigating the physicochemical properties of the phytochemicals. Pharmacokinetics properties included Blood Brain Barrier (BBB) permeability, Human Intestinal absorption (HIA), P-glycoprotein (PGP), and five major isoforms from the cytochromes P450 (CYP1A2, CYP2C19, CYP2C9, CYP2D6, CYP3A4) [76,77]. For toxicity analysis, AMES toxicity, hERG (human ether-a-go-go related gene) inhibition, human hepatotoxicity, and rat toxicity (LD50) were considered. Non-bonded interactions of the *ctxB*-phytochemical complexes, including their categories and types, were visualized using Discovery Studio Visualizer [78].

### 2.5 Molecular dynamics (MD) simulation

Molecular dynamics simulations were executed to evaluate the stability of *ctxB*-phytochemical complexes by using YASARA software [79]. The initial MD simulation run was executed for 100 ns using YASARA md_run.mcr script (https://www.yasara.org/md_run.mcr). In the script, the default AMBER14 (Assisted Model Building with Energy Refinement) force field [80] was utilized to determine the dynamic behavior of the *ctxB*-phytochemical complexes, and the TIP3P (Transferable Intermolecular Potential 3 Points) water model [81] was applied to optimize the hydrogen bonding network of the complexes. A water density of 0.997 g/ml was considered to maintain periodic boundary conditions. The simulation took place in a cubic virtual environment with default physiological conditions (pH of 7.4, the ion concentration of 0.9% NaCl, 298 K temperature, and constant pressure), and long-range Coulomb forces [82,83]. The initial energy of each complex was minimized using simulated annealing with the steepest gradient approach over 5,000 cycles. After minimization, a built-in macro script (https://www.yasara.org/md_run.mcr) of YASARA was used to equilibrate the system under controlled conditions to reach stable temperature, pressure, and density. Equilibration was performed under the NVT ensemble, where the temperature was gradually brought to the target (298K) using a Berendsen thermostat [84], and the solvent density was maintained at a constant level (thereby keeping the pressure effectively constant) using Densostat [82]. Simulation snapshots were stored every 250 ps (fast step) in sim format (.sim), and the trajectories were captured. The Root mean square deviation of protein backbone (RMSDBb), ligand movement RMSD, ligand conformation RMSD, radius of gyration (Rg), Root mean square fluctuations (RMSF), number of hydrogen bonds, per-residue protein secondary structure changes, per-residue contact with ligand, and protein secondary structure content were analyzed using the md_analyze.mcr script (https://www.yasara.org/md_analyze.mcr). The analyzed results were visualized by custom Python scripts. In addition, MD simulations for all of the complexes were also conducted using GROMACS and compared with the results from YASARA (S1 File).

### 2.6 Binding free energy and per-residue energy decomposition

Binding energies were calculated using MM-Poisson-Boltzmann surface area (MM-PBSA) with the AMBER14 force field [80] at 298K in a cuboid cell using YASARA (https://www.yasara.org/md_analyzebindenergy.mcr) [79,85,86]. The software has used the following formula to calculate binding energy:


$$\text{BindingEnergy}=\;{\text{E}}_{\text{potRecept}}+{\text{E}}_{\text{solRecept}}+{\text{E}}_{\text{potLigand}}+{\text{E}}_{\text{solvLigand}}-{\text{E}}_{\text{potComplex}}-{\text{E}}_{\text{solvComplex}}$$

Here, E_pot_ denotes gas-phase molecular mechanics potential energy, including bonded, electrostatic, and van der Waals interactions, and E_solv_ denotes total solvation energy, consisting of both polar (Poisson-Boltzmann) and non-polar (Surface accessible surface area-based) contributions. E_potComplex_ and E_solvComplex_ refer to the potential energy and solvation energy of the total complex. E_potRecept_ and E_solRecept_ refer to the potential energy and solvation energy of the receptor. E_potLigand_ and E_solvLigand_ refer to the potential energy and solvation energy of the ligand.

Per-residue energy decomposition analysis was performed to quantify the energetic contribution of amino acids involved in ctxB-phytochemical complexes. For this, all complexes were subject to 100 ns MD simulations using GROMACS [87], and the resulting trajectories were analyzed (see S1 File). g_mmpbsa tool [88] was used for residue-level energy decomposition. This tool first decomposes the overall MM-PBSA binding energy of the protein-ligand complex, then calculates the contributions of each residue. MM-PBSA binding free energy was also calculated using the g_mmpbsa tool (see S1 File). Moreover, backbone RMSD (RMSDBb), ligand movement RMSD, ligand conformation RMSD, radius of gyration (Rg), RMSF, and hydrogen bond counts were obtained from both YASARA and GROMACS.

## 3. Results

### 3.1 Structure prediction of the *ctxB* mutants

**Fig 1:**
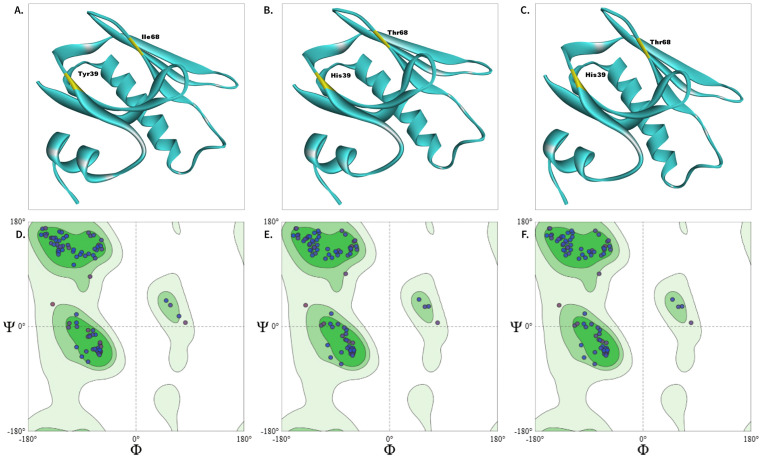
Predicted structures and Ramachandran plots for ctxB genotypes. Panels (A-C) show the predicted 3D structures of *ctxB3* (A), *ctxB1* (B), and *ctxB7* (C) using SWISS-MODEL. Mutant positions were highlighted in yellow, and corresponding amino acids were highlighted in the plots. Quality of the predicted structures was evaluated using Ramachandran plots (D-F). In the plots, confidence regions were visualized using three different green color intensities for referring to highly favored (green color shade), allowed (light green color shade), and generously allowed (extra-light green color shade) backbone φ and Ψ dihedral angles of each amino acid residue in protein structures. Most of the amino acid residues within the high (under green polygon) and moderate (under light-green polygon) confidence regions indicate the reliability and structural validity of the predicted models.

Three genotypes of the ctxB gene (*ctxB1*, *ctxB3*, and *ctxB7*) have been observed in the 7^th^ pandemic *V. cholerae* O1 El Tor strains, which are characterized by three-point mutations. All of the genotypes have a total of 124 amino acids (S2 Dataset). *ctxB3* differs from *ctxB1 by* two amino acid substitutions: HIS39 replaced TYR39, and THR68 replaced ILE68, whereas *ctxB7* has an extra single nucleotide change within the signal peptide region (1−21 amino acids), ASN20 replaced HIS20. The predicted 3D structures of the proteins encoded by three distinct ctxB genotypes were displayed in Fig 1A-C, and prediction details are provided in S1 Table. Signal peptide region (first 21 amino acids) of the target protein (ctxB genotypes) didn’t have an equivalent crystal structure. Most of the amino acid residues for the predicted structures were located within the high (under green polygon) and moderate (under light-green polygon) confidence regions of phi (φ) and psi (ψ) dihedral angles in the Ramachandran plot, indicating the structural validity of the predicted models (Fig 1D-F). TM-align was used to observe the differences between the predicted structures of three ctxB genotypes [89]. *ctxB1* and *ctxB7* were highly similar (RMSD-0, sequence identity-1.0), while both showed slight differences from *ctxB3* (RMSD-0.26, sequence identity-0.981).

### 3.2 Screening of phytochemicals by molecular docking analysis

Molecular docking was employed to calculate the binding affinity scores (kcal/mol) of the selected 7,607 phytochemicals against proteins encoded by three genotypes of *ctxB* (*ctxB1*, *ctxB3*, and *ctxB7*). A binding affinity of less than −7 kcal/mol is generally considered indicative of strong affinity binding [90]. In this study, we used a slightly higher cutoff for binding affinity (≤ −7.5 kcal/mol) to identify high-confidence interactions. A total of 298 phytochemicals were shortlisted based on the binding affinity cutoff, of which 101 were from *Morus alba*, 24 from *Citrus aurantium*, 17 from *Emblica officinalis*, 16 from *Capsicum annuum*, 15 from *Psidium guajava*, *and* 125 phytochemicals from 32 other plants (S3 Dataset and S2 Table).

### 3.3 ADME/T analysis

**Fig 2:**
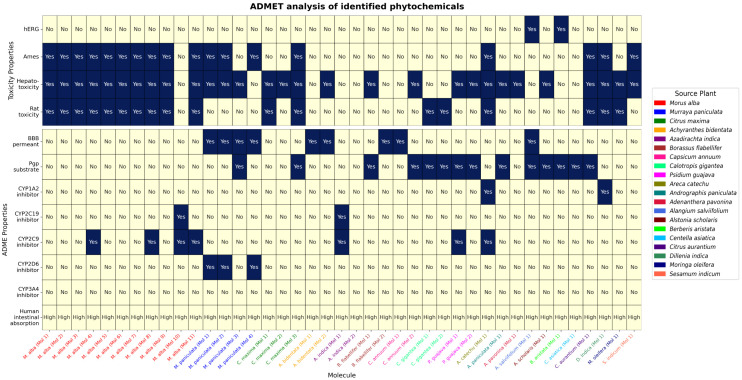
ADME/T analysis results of the 41 candidate phytochemicals. Horizontal axis shows the re-coded name of the phytochemicals (with source plant name), and different color is used for highlighting source plants. Vertical axis shows different measurements for the ADME and toxicity properties. Dark blue color in the plot indicates presence, and light yellow indicates absence in a specific test result. Seven phytochemicals that satisfied for ADME/T criteria were considered in the subsequent analyses.

ADME/T analysis was performed to investigate the pharmacokinetic properties of the selected phytochemicals with docking score ≤ −7.5 kcal/mol. Out of 298 phytochemicals, 87 satisfied physicochemical properties, such as lipophilicity (ref. range: −0.7 < XLOGP3 < +6.0), topological polarity surface area (ref. range: 20Å² < TPSA < 140Å²), solubility (ref. range: log S not higher than 6), and rotatable bonds (ref. ≤ 9). In addition, optimum physicochemical properties were observed for another 16 phytochemicals with only little higher lipophilicity (obs. range: + 6.0 < XLOGP3 < +6.8) (S4 Dataset). Among the total 103 (n = 87 + 16) phytochemicals with optimum physicochemical properties, 41 satisfied several additional pharmacokinetic properties, including Blood Brain Barrier (BBB), Human Intestinal Absorption (HIA), P-glycoprotein (PGP), and the activity of five major isoforms (CYP1A2, CYP2C19, CYP2C9, CYP2D6, CYP3A4) (Fig 2). Phytochemicals with satisfactory ADME properties can be extracted from *M. alba* (n = 11), *M. paniculata* (n = 4), *C. maxima* (n = 3), and several other plants (n = 2 × 6 + 1 × 11 = 23) (S2 Table). Note that the multiple candidate phytochemicals from the same plant source were recoded as Mol 1, Mol 2, etc., with a prefix of the respective plant name to further include them in the downstream analyses (S3 Table).

After evaluating the ADME properties, toxicity properties were tested based on four different parameters, such as hERG inhibition, AMES toxicity, hepatotoxicity, and rat toxicity. Among 41 phytochemicals, 14 showed AMES positivity, hepatotoxicity, and rat toxicity, 3 showed AMES and hepatotoxicity, 2 showed hepatotoxicity and rat toxicity, and 15 others showed toxicities in at least one of four parameters. Remaining seven phytochemicals of the plants, *M. alba* (Mol 10), *A. indica* (Mol 1 and Mol 2), *A. bidentata* (Mol 1), *B. flabellifer* (Mol 2), *C. annuum* (Mol 1), and *C. asiatica* (Mol 1), passed in toxicity analysis.

### 3.4 Binding sites comparisons between the ctxB-host receptor and the ctxB-phytochemical complexes

**Fig 3:**
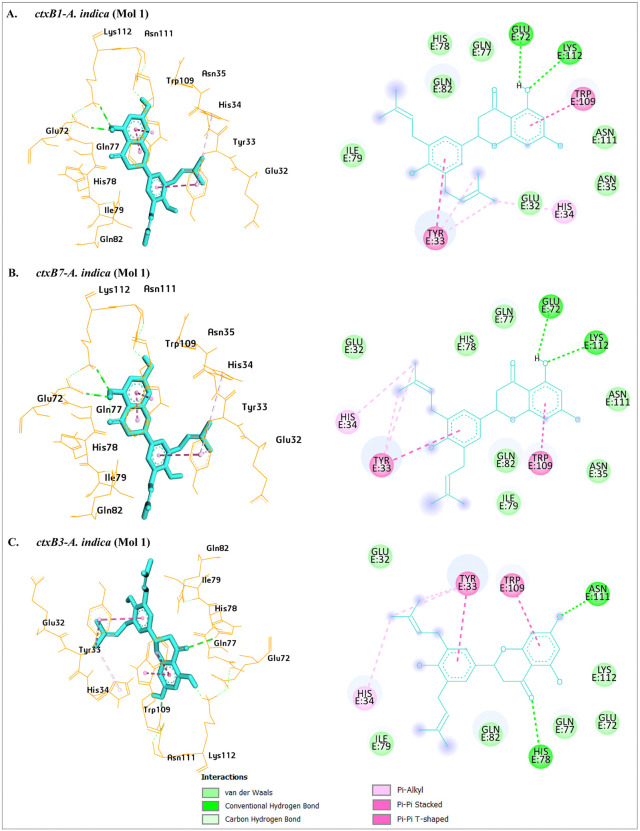
3D and 2D visualizations of the ctxB*-*A. indica (Mol 1) complexes. Here, 3D and 2D views of the complexes were visualized on the left and right sides of each figure, respectively. In the 3D view, orange-colored structures represent amino acids of ctxB, greenish-blue-colored structures represent phytochemicals, and dashed lines indicate hydrogen and hydrophobic bonds. In the 2D view, two different types of green circles (dark and extra-light) represent amino acids involved in hydrogen bonds, such as, dark green circle refers to conventional hydrogen bonds, and extra-light green circle refers to carbon-hydrogen bonds. Textualized forms of amino acid and positions highlighted by light green color represent van der Waals forces. Pink circle represents amino acids involved in hydrophobic bonds, and its different color intensity refers to hydrophobic bond types, such as dark pink refers to pi-pi stacked and pi-pi T-shaped bond, and light pink refers to pi-alkyl bond. A phytochemical of *A. indica* (Mol 1) interacted with *ctxB* genotypes through 11–12 amino acid residues, all of which are common for the *ctxB*-GM1 complex.

Since cholera disease starts with the binding of ctxB to host receptors, observing the binding positions may provide valuable insights into the probable inhibitory properties. Cholera toxin can be inhibited at two different stages: (1) before being secreted, proper folding of the *ctxB* pentamer in the bacterial cytoplasm or periplasm can be prevented during the intracellular assembly [91], and (2) host cell binding stage: the secreted *ctxB* pentamer can be prevented from binding to the host receptors [38,39]. We searched for published literature to investigate the binding positions of the ctxB pentamer with two different receptors (GM1 and histo-blood group antigen Lewis^x^ (Le^x^)). The primary receptor GM1 pentasaccharides mainly bind with *ctxB* (classical, similar to *ctxB1*) at GLU32, TYR33, HIS34, GLU72, HIS78, ILE79, GLN82, TRP109, ASN111, and LYS112 residues [92]. Binding positions of the *ctxB* and histo-blood group antigen Lewis^x^ (Le^x^) complex, and the ctxB pentamer itself, were tabulated to identify active amino acid residues [93–95] (S4 Table). Binding behaviors of *ctxB* and the 7 candidate phytochemicals (docking score ≤ −7.5 kcal/mol and passed in ADME/T analysis) were further investigated to understand their potential inhibiting properties. All the possible (3 × 7 = 21) complexes of potential ligands with three *ctxB* genotypes were analyzed, and it was observed that most of the phytochemicals (n = 6) interacted with several common amino acid residues (≥ 6) at which host receptors interact (S5 Dataset). Interestingly, a phytochemical from *A. indica* constructed bonds with *ctxB* at 11 amino acid residues, all of which are common to the binding residues of the *ctxB*-GM1 complex (Fig 3A-C). Similarly, a phytochemical from *M. alba and ctxB* complexes (three complexes for three different genotypes) produced 7–8 common amino acid residues to the ctxB pentamer interacting binding residues.

### 3.5 Intermolecular interactions of *ctxB* and phytochemical complexes

We further investigated types of chemical interactions in the *ctxB*-phytochemical complexes. Analysis showed two hydrogen bonds, six hydrophobic bonds, and seven van der Waals forces in *ctxB1*-*A. indica* (Mol 1) complex. Two hydrogen bonds (conventional) were at the LYS112, GLU72, the six hydrophobic bonds were at TRP109 (two pi-pi stacked), TYR33 (pi-pi T-shaped), TYR33 (two pi-alkyl), HIS34 (pi-alkyl), and seven van der Waals forces at GLU32, ASN35, GLN77, HIS78, ILE79, GLN82, and ASN111 (Fig 3A). *ctxB7*-*A. indica* (Mol 1) complex showed identical interactions as *ctxB1*-*A. indica* (Mol 1) complex (Fig 3B). Slight variations in binding types and positions were observed for *ctxB3*-*A. indica* (Mol 1) complex (Fig 3C). Similarly, chemical interactions and binding types of the other four candidate phytochemicals were listed in Table 1, and the 2D views of these complexes were visualized in S1 Fig.

**Table 1:** The non-bonded interaction of phytochemicals and ctxB genotypes.

Molecule	ctxB genotypes (Docking score)	Hydrogen bond		Hydrophobic bond and others		Van-der waals force
		*ctxB* amino acid position	Bond Category	*ctxB* amino acid position	Bond Category	*ctxB* amino acid position
*A. indica* (Mol 1) PubChem ID- 442153	*ctxB7* (−8 kcal/mol)	LYS112	Conventional	TRP109 (2)	Pi-Pi Stacked	LEU79, HIS78, GLN82, GLN77, GLU32, ASN111, ASN35
		GLU72	Conventional	TYR33	Pi-Pi T-shaped	
				TYR33 (2)	Pi-Alkyl	
				HIS34	Pi-Alkyl	
	*ctxB1* (−8 kcal/mol)	LYS112	Conventional	TRP109 (2)	Pi-Pi Stacked	ILE79, HIS78, GLN82, GLN77, GLU32, ASN111, ASN35
		GLU72	Conventional	TYR33	Pi-Pi T-shaped	
				TYR33 (2)	Pi-Alkyl	
				HIS34	Pi-Alkyl	
	*ctxB3* (−7.5 kcal/mol)	HIS78	Conventional	TRP109 (2)	Pi-Pi Stacked	ILE79, LYS112, GLN82, GLN77, GLU32, GLU72
		ASN111	Conventional	TYR33	Pi-Pi T-shaped	
				TYR33 (2)	Pi-Alkyl	
				HIS34	Pi-Alkyl	
*A. bidentata* (Mol 1) PubChem ID- 99474	*ctxB7* (−7.8 kcal/mol)	ASN111	Conventional	ILE79	Alkyl	GLN82, GLN77, GLU72, HIS34, LYS112
				TYR33	Pi-Alkyl	
				TRP109 (6)	Pi-Alkyl	
	*ctxB1* (−7.8 kcal/mol)			ILE79	Alkyl	GLN82, GLN77, GLU72, HIS34, LYS112
				TYR33	Pi-Alkyl	
				TRP109 (5)	Pi-Alkyl	
	*ctxB3* (−7.9 kcal/mol)	GLU72	Conventional	ILE79	Alkyl	GLN82, GLN77, ASN111, HIS78, LYS112
				TYR33	Pi-Alkyl	
				TRP109 (4)	Pi-Alkyl	
				HIS34	Pi-Alkyl	
*B. flabellifer* (Mol 2) PubChem ID- 441900	*ctxB7* (−7.8 kcal/mol)			ILE79	Pi-Alkyl	HIS34, GLN77, GLN82, GLU72, ASN111, LYS112
				TYR33	Alkyl	
				TRP109 (6)	Pi-Alkyl	
	*ctxB1* (−7.8 kcal/mol)	LYS112	Conventional	TRP109 (4)	Pi-Alkyl	HIS34, GLN77, GLN82, GLU72
		ASN111	Conventional	ILE79	Alkyl	
				TYR33	Pi-Alkyl	
	*ctxB3* (−7.9 kcal/mol)	GLU72	Conventional	TRP109 (4)	Pi-Alkyl	GLN82, GLN77, ASN111, HIS78, LYS112
				ILE79	Alkyl	
				TYR33	Pi-Alkyl	
				HIS34	Pi-Alkyl	
*M. alba* (Mol 10) PubChem ID- 145955804	*ctxB7* (−7.8 kcal/mol)	ARG94	Conventional	GLU50	Pi-Anion	GLU57, SER51, MET58, ALA59, TYR48, ASP91
		GLU87	Conventional	GLU87	Pi-Anion	
				PRO74 (2)	Alkyl	
				VAL73	Alkyl	
				LEU52 (2)	Alkyl	
				LYS90	Pi-Alkyl	
	*ctxB1* (−7.8 kcal/mol)	LYS90	Conventional	GLU50	Pi-Anion	GLU57, SER51, MET58, ALA59, TYR48, ASP91
		ARG94	Conventional	GLU87	Pi-Anion	
				PRO74 (2)	Alkyl	
				VAL73	Alkyl	
		GLU87	Conventional	LEU52 (2)	Alkyl	
				LYS90	Pi-Alkyl	
	*ctxB3* (−7.5 kcal/mol)	GLN82	Conventional	GLN82	Pi-Sigma	HIS78, VAL71, MET89, SER81
		ILE117	Conventional	ALA85	Alkyl	
				ILE79	Alkyl	
				TYR33	Pi-Alkyl	
		GLU72	Conventional	TRP109	Pi-Alkyl	
				ALA85	Pi-Alkyl	
				ILE86	Pi-Alkyl	
*A. indica* (Mol 2) PubChem ID- 52951893	*ctxB7* (−7.5 kcal/mol)	HIS34 (2)	Conventional	TRP109 (2)	Pi-Pi Stacked	GLU72, HIS78, GLN82, TYR33, GLN77, ASN35, LYS112
		ASN111	Conventional			
		TRP109	Carbon			
	*ctxB1* (−7.5 kcal/mol)	HIS34 (2)	Conventional	TRP109 (2)	Pi-Pi Stacked	GLU72, HIS78, GLN82, TYR33, GLN77, ASN35, LYS112
		ASN111	Conventional			
		TRP109	Carbon			
	*ctxB3* (−7.5 kcal/mol)	ILE120	Conventional	MET89	Pi-Sulfur	TYR33, ALA118, TRP109, ILE86, ALA119, LEU29, SER81
		ARG88	Carbon	ALA85	Pi-Alkyl	
		THR92	Carbon			
		GLN82	Pi-Donor	ARG88	Pi-Alkyl	

In the table, molecule refers to the candidate phytochemical, which was presented along with its PubChem ID. Docking scores of *ctxB*-phytochemical complexes, hydrogen and hydrophobic bond types with binding positions, and nonchemical binding positions with van der Waals force were tabulated. Hydrogen bonds include conventional, hydrogen-carbon, and pi-donor. Hydrophobic bonds include alkyl, pi-alkyl, pi-pi stacked, pi-sulfur, and pi-sigma. Other bonds include the electrostatic bonds (pi-anion) and pi-sulfur.

### 3.6 Molecular dynamics simulations

**Fig 4:**
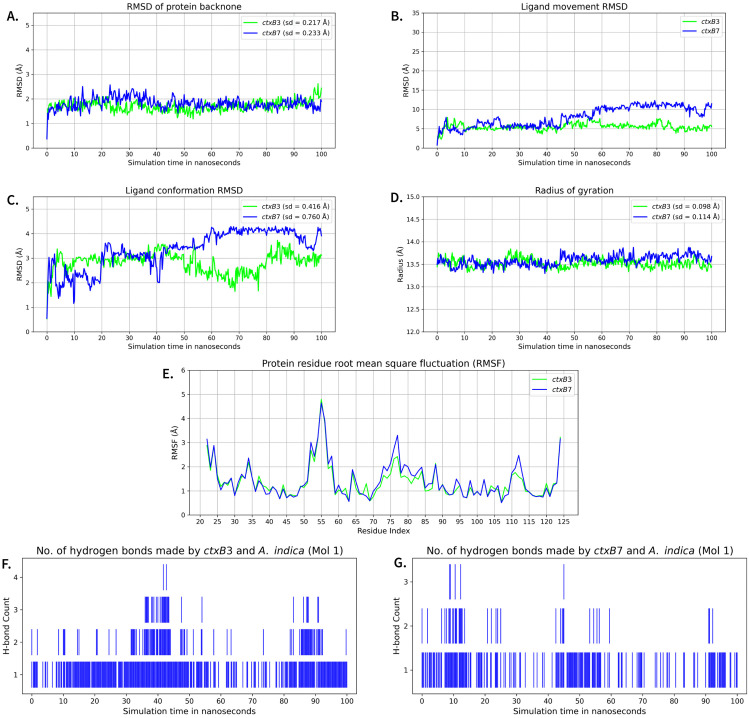
Molecular dynamics simulation results for *ctxB3*-*A. indica* (Mol 1) and *ctxB7*-*A. indica* (Mol 1) complexes. (A) RMSD of protein backbone, (B) ligand movement RMSD, (C) ligand conformation RMSD, (D) radius of gyration, (E) protein residue root mean square fluctuation (RMSF), (F) number of hydrogen bonds in *ctxB3*-*A. indica* (Mol 1) complex, and (G) number of hydrogen bonds in *ctxB7*-*A. indica* (Mol 1) complex. In each of the plots, the horizontal axis represents simulation time (in ns), and the vertical axis represents the measured values of different analyses, except for (E), where the horizontal axis represents the residue index. The green line indicates *ctxB3*-*A. indica* (Mol 1) complex, and the blue line indicates *ctxB7*-*A. indica* (Mol 1) complex. The protein backbone RMSD and radius of gyration are stable, and RMSF fluctuates in the coil/turn regions. Ligand movement RMSD increased gradually for *ctxB7*-*A. indica* (Mol 1) complex, indicated positional rearrangement within the binding site, while the ligand conformation RMSD reflected substantial internal structural adjustments.

Molecular dynamics simulations were conducted using YASARA for 100 ns to observe variations in protein backbone RMSD, ligand movement RMSD, ligand conformation RMSD, radius of gyration, protein residue root mean square fluctuation (RMSF), number of hydrogen bonds, per-residue protein secondary structure changes, per-residue contact with ligand, and overall protein secondary structure contents for different complexes. In simulation, minor variations were observed for each of the phytochemicals and two different targets (*ctxB3* and *ctxB7* genotypes). For example, analysis results for *A. indica* (Mol 1) were very similar for the complexes with *ctxB3* and *ctxB7*. Protein backbone RMSD and radius of gyration were quite stable after initial equilibration for both *ctxB3*-*A. indica* (Mol 1), and *ctxB7*-*A. indica* (Mol 1) complexes, reflecting the stability of overall protein folding and its compactness throughout the 100 ns simulation (Fig 4A, D). In *ctxB3*, ligand movement RMSD remained relatively low (~6 Å), and conformational RMSD around ~3 Å, suggesting that the ligand remained largely within the initial binding pocket while adapting its internal conformation (Fig 4B, C). In contrast, the ligand bound to *ctxB7* exhibited an increase in movement RMSD from ~5 Å during the initial 45 ns to ~11 Å thereafter, conformational RMSD also increased from ~3 Å to ~4 Å after 45 ns, indicating that the ligand may have explored different binding pockets to exhibit more structural flexibility of the ligand (Fig 4B, C). *A. indica* (Mol 1) interact with ctxB through mostly hydrophobic bonds (S6E, F Fig), whereas, only a hydrogen bond was observed in almost all of the time points for *ctxB3-A. indica* (Mol 1) complex, but was rare for the *ctxB7-A. indica* (Mol 1) (Fig 4F, G). For these complexes, β-sheet content (~38–42%) and α-helix content (~24–28%) were stable at overall and residue levels, whereas turn and coil regions fluctuated moderately throughout the simulation (S6A-D Fig). The higher RMSF values were mainly observed in two regions, residues 50–57 and 76–78 (Fig 4E). Similarly, all other phytochemical-ctxB complexes showed comparable behavior throughout the simulation (S6-S10 Fig). Interestingly, ligand movement RMSD was generally higher for all complexes, although *B. flabellifer* (Mol 2), *A. bidentata* (Mol 1), and *A. indica* (Mol 2) did not exhibit significant conformational changes and remained stable in their initial structural forms (S2, S4-S5 Fig). Simulation analysis using GROMACS yielded similar results for the complexes (S11-S15 Fig).

### 3.7 MM-PBSA binding free energy

The MM-PBSA method was employed to estimate the binding free energies of all ctxB-phytochemical complexes using two software packages, YASARA and GROMACS. The calculated average binding energies of each complex with individual energy components are shown in Table 2. The average binding energies calculated using YASARA were favorably higher (positive scale for favorable binding), ranging between 93.58 and 152.06 kJ/mol, except for *ctxB7-M. alba* (mol 10) complex (average binding energy = 7.17 kJ/mol). The binding energies of this complex were relatively low during the initial 25 ns, increased between 25 and 50 ns, again decreased between 50 and 70 ns, and subsequently stabilized at higher values, with an average binding energy of 66.191 kJ/mol between 70 ns and 100 ns (S16 Fig). On the other hand, GROMACS results showed that two phytochemicals from *A. indica* exhibited comparatively more favorable binding energies (negative scale for favorable binding) to both ctxB variants, whereas *M. alba* (mol 10) showed the least favorable energies. Most of the complexes displayed large negative VdW energies (−70.868 to −132.813 kJ/mol), indicating hydrophobic interactions were the main stabilizers. Nonpolar solvation energy (SASA) was also favorable for the complexes (−9.887 to −17.305 kJ/mol). The *ctxB7-A. indica* (mol 1) complex showed a strong electrostatic energy contribution (−47.633 kJ/mol), whereas it was relatively smaller in other complexes. Polar solvation energies were positive (38.071 to 99.500 kJ/mol), reducing net binding. However, both YASARA and GROMACS showed favorable average binding energies for all of the complexes (S16 Fig).

**Table 2:** The MM-PBSA binding free energy by YASARA and GROMACS.

Complex	VdW	Elec	Pol	SASA	Average binding energy (GROMACS)	Average binding energy (YASARA)
*ctxB3-A. indica* (mol 1)	−103.921	−25.046	54.016	−13.536	−88.487	93.58
*ctxB7-A. indica* (mol 1)	−113.68	−47.633	94.187	−16.16	−83.281	115.58
*ctxB3-A. bidentata* (mol 1)	−75.486	−12.709	56.276	−10.451	−42.370	150.95
*ctxB7-A. bidentata* (mol 1)	−70.868	−11.167	38.071	−9.887	−53.852	106.64
*ctxB3-B. flabellifer* (mol 2)	−84.510	−9.399	46.613	−10.768	−58.064	152.06
*ctxB7-B. flabellifer* (mol 2)	−75.875	−6.690	47.025	−10.446	−45.986	112.90
*ctxB3-M. alba* (mol 10)	−77.811	−31.726	99.500	−10.402	−20.438	106.44
*ctxB7-M. alba* (mol 10)	−85.409	−15.629	90.495	−11.682	−22.225	7.17
*ctxB3-A. indica* (mol 2)	−121.311	−31.214	81.609	−15.159	−86.075	142.37
*ctxB7-A. indica* (mol 2)	−132.813	−15.065	80.279	−17.305	−84.904	129.47

Here, all the energy components were calculated in kJ/mol. VdW means Van der Waals force contribution, Elec means electrostatic energy contribution, Pol means polar solvation energy, and SASA means nonpolar solvation energy. Note, YASARA calculates favorable binding energy in positive scale, and GROMACS calculates in negative scale.

### 3.8 Per-residue binding energy decomposition

**Fig 5:**
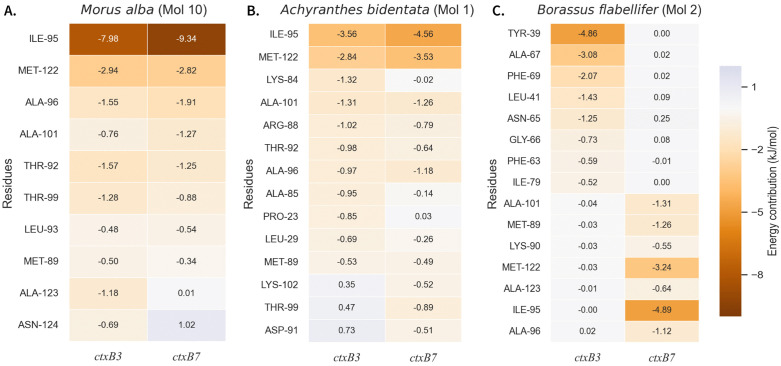
Per residue binding free energy decomposition of *ctxB-*phytochemical complexes. Panel A shows *Morus alba* (Mol 10), Panel B shows *Achyranthes bidentata* (Mol 1), and Panel C shows *Borassus flabellifer* (Mol 2). In each of the panels, the horizontal axis represents ctxB variants (*ctxB3*, *ctxB7*), and the vertical axis represents the positions of the amino acid residues. Orange color indicates negative (favorable) values, purple indicates positive (unfavorable) values, and white denotes near-zero contributions. For both *ctxB3* and *ctxB7*, *M. alba* (Mol 10) and *A. bidentata* (Mol 1) shared a comparable profile of major amino acid contributors to the total binding energy.

Per-residue binding energy decomposition was performed to evaluate the contributions of individual amino acid residues of ctxB in binding. It was observed that mostly different sets of high contributing residues for *ctxB3* and *ctxB7*, when its interact with the same phytochemical. For example, binding of *A. indica* (mol 1) with *ctxB3*, ILE60 (−11.214kJ/mol), ILE68 (−4.357 kJ/mol), THR49 (−4.281 kJ/mol), PRO114 (−4.277 kJ/mol), MET58 (−3.377 kJ/mol), THR62 (−2.318 kJ/mol), and GLU50 (−2.283 kJ/mol) were the top contributors*,* whereas, TRP109 (−4.739 kJ/mol), MET89 (−3.563 kJ/mol), TYR33 (−2.425 kJ/mol), ILE86 (−2.346 kJ/mol), and LEU29 (−2.176 kJ/mol) were the top contributors when the phytochemical binds with *ctxB7*. We obsevered exceptions only for *M. alba* (Mol 10) and *A. bidentata* (Mol 1), whereas similar set of residues of *ctxB3* and *ctxB7* contributed largely (Fig 5A-B, S17 Fig). All residues contributing moderately (≤−0.2 kJ/mol) to strongly (≤−11.214 kJ/mol) to the binding energy for all of the complexes were tabulated in S6 Dataset. Amino acid residues collectively account for ~80% of the total binding energy, and individual contribution ≤ −0.5 kJ/mol were listed and visualized (S18 Fig). A total of 70 out of 124 amino acid residues were found as high contributors for the different complexes.

### 3.9 Intermolecular interactions after simulation

Binding residues of the complexes were reobserved after simulation. The total non-bonded interactions and interacting residues increased in almost all complexes except for the *ctxB7*-*M. alba* (Mol 10) (interacting residues decreased from 13 to 8), and *ctxB7*-*A. bidentata (Mol 1)* (interacting residues decreased from 9 to 8) complexes. Each complex changed some of its interacting residues after simulation. A detailed list of all the non-bonded interactions before and after the simulation was provided in S7 Dataset. It was noted that the complexes showed an increasing number of common residues with the pentamer-interacting residues after simulation. However, exceptions were for Mol 1 and Mol 2 of *A. indica*, and for these interacting residues of ctxB-phytochemical were mostly common to the interacting residues of ctxB-GM1 complex.

## 4. Discussion

Antimicrobial resistance is often referred to as a silent pandemic, which would be responsible for taking millions of lives in the near future [96,97]. Increasing trend of multidrug resistance to the existing antibiotics represents a growing challenge for future treatment strategies, highlighting the need for alternative therapeutic approaches. Medicinal plants/herbs have been utilized as a source of many traditional medicines since ancient times, and still serve as a primary source of novel drugs in the pharmaceutical industry [98]. With the advantages of recent technological advancements, the identification and validation of natural products may aid in their effective use and repurposing for treating different diseases. This study conducted in-silico identification and validation of plant-derived phytochemicals for treating cholera disease by targeting cholera toxin with the goal of complementing current cholera treatment strategies. Pathogenic *V. cholerae* strains carry CTX prophage gene cassette, including cholera toxin-encoding genes [99,100]. The mechanism of cholera toxin (ctxAB) initiates with the high-affinity attachment of its B subunits to host monosialoganglioside GM1 receptors or through comparatively low-affinity binding to secondary receptors [34–42]. Thus, inhibiting this initial interaction of the toxin could prevent the pathogenicity of *V. cholerae* infection and the onset of cholera [101–107]. With genomic evolutionary progression of the 7PET *V. cholerae* O1 El Tor strains over time, three genotypes of ctxB (*ctxB1*, *ctxB3*, and *ctxB7*) were observed. A previous study showed the variations in the binding ability of different genotypes of ctxB to GM1 [108]. Therefore, in this study, proteins encoded by three different genotypes of ctxB were considered as targets. The resource of IMPPAT database served as primary base for including a list of 7,607 phytochemicals incorporating historical information of the corresponding 55 source plants used for treating cholera. After subsequent molecular docking analysis of the phytochemicals, 298 potential compounds were identified based on high binding affinity (≤ −7.5 kcal/mol), which can be sourced from 37 medicinal plants, including *Morus alba, Citrus aurantium, Emblica officinalis, Capsicum annuum,* and *Psidium guajava.* Further drug-likeness and pharmacokinetics analysis reduced candidates to 41 phytochemicals, of which seven phytochemicals satisfied toxicity properties. Among these, six phytochemicals interacted with known active residues of *ctxB*, and one of which, from *Capsicum annuum*, has previously been reported to have toxic effects [109,110]. The remaining five phytochemicals, such as *A. indica* (Mol 1), *A. bidentata* (Mol 1), *B. flabellifer* (Mol 2), *M. alba* (Mol 10), and *A. indica* (Mol 2), were further analyzed for understanding their binding stability with ctxB. Molecular dynamics simulations over 100 ns showed stability in backbone RMSD and radius of gyration, which indicate global structural stability and compactness of protein-ligand complexes. Secondary structure analyses further confirmed the maintenance of α-helix and β-sheet elements throughout the simulation. In contrast, analyses of ligand movement RMSD and ligand conformational RMSD clearly showed that all of the phytochemicals shifted from their initial binding pockets, with some exhibiting internal conformational changes. These trends were also consistently observed in GROMACS analysis results. Additionally, minor secondary structure transitions occurred in flexible turn and coil regions, suggesting that these regions accommodate ligand-induced conformational adjustments. The higher RMSF values were also observed for all complexes in the coil and turn regions, reflecting their inherent flexibility and potential role in ligand binding. The total number of hydrogen bonds and per-residue ligand contact analyses revealed that hydrophobic interactions were the main driving force for the binding of the complexes. Most of the complexes exhibited favorable MM-PBSA binding free energies in both YASARA and GROMACS simulations. GROMACS results indicated that *M. alba* (mol 10) had the least favorable binding (less negative) with ctxB genotypes among the five phytochemicals. The large contribution of van der Waals energies to total binding energy for all complexes further highlighted the importance of hydrophobic interactions in complex stabilization. Per-residue energy decomposition revealed that different complexes were stabilized by distinct sets of amino acids, collectively reflecting a diverse pattern of interactions. Intermolecular interactions after YASARA simulations showed that all phytochemicals altered their initial interacting residues, whereas *A. indica* (Mol 1), *A. indica* (Mol 2) maintained significant interactions with GM1-binding residues.

Although phytochemicals were listed in this study based on specific plants, a phytochemical can be found in several plants. For example, a phytochemical named *A. indica* (Mol 1) in this study, originally known as Abyssinone V, a prenylated flavonoid, can be found in several plants, such as *Erythrina abyssinica, Azadirachta indica* (neem). In addition, the candidate phytochemicals identified in this study have also been reported in previous studies for treating other diseases. For example, Abyssinone V (named in this study as *A. indica* (Mol 1)) has been reported as an inhibitor of the varicella-zoster virus [111]. Another phytochemical named *A. bidentata* (Mol 1), originally known as Diosgenin, a steroidal sapogenin, has been shown as beneficial for cognitive function and useful for treating several diseases [112]. *B. flabellifer* (palm) (Mol 2), originally known as Yamogenin, is a diastereomer of diosgenin, reported as an inhibitor of lipid accumulation in hepatocytes [113]. The other two phytochemicals, *M. alba* (Mol 10) and *A. indica* (Mol 2), have not been reported yet.

## 5. Conclusion

This study explored the potential phytochemicals that can be extracted from the medicinal plants historically used for treating cholera in the Indian subcontinent, providing theoretical relevance of using those plants and alternative medications for treating the disease. We investigated the binding behaviors of the phytochemicals with ctxB and identified toxin-inhibitory properties for a large set of phytochemicals. However, many compounds were identified as toxic, and only five from 7,607 phytochemicals were found to be as most promising candidates for inhibiting cholera toxin. Now, further in vitro and in vivo experimental studies are required to evaluate the effectiveness of the candidate phytochemicals.

## Supporting information

S1 File Supplementary methods for GROMACS simulations. Detailed descriptions of system preparation, simulation parameters, equilibration, production runs, and trajectory analyses (e.g., RMSD, RMSF, Radius of gyration, number of hydrogen bonds, MM-PBSA binding energy) were performed using GROMACS.
(DOCX)

S1 Table Structural prediction details of ctxB toxin alleles using SWISS-MODEL. Model quality metrics (QSQE, GMQE), and template information for ctxB alleles are provided.
(DOCX)

S2 Table Plant-specific phytochemicals at different screening steps. Distribution of phytochemicals from individual plant sources across successive screening steps, including molecular docking, drug-likeness, and pharmacokinetic filtering.
(DOCX)

S3 Table Phytochemicals with recoded names. Original phytochemical names, their PubChem ID, and corresponding recoded names are listed.
(DOCX)

S4 Table The active residues of ctxB. Key amino acid residues involved in ligand binding and toxin functionality are reported.
(DOCX)

S1 Dataset Total phytochemicals used in the study. A total of 7,607 phytochemicals extracted from 54 medicinal plants are listed, with their PubChem ID or source publications and corresponding source plant.
(XLSX)

S2 Dataset Protein sequence of ctxB genotype. Protein sequences and sequence IDs of three ctxB genotypes (*ctxB1*, *ctxB3*, and *ctxB7*) are provided. All of the genotypes comprise 124 amino acids. Compared with *ctxB1*, *ctxB3* contains two amino acid substitutions (HIS39 ◊ TYR39, and THR68 ◊ ILE68, whereas *ctxB7* differs by a single nucleotide change within the signal peptide region (residues 1–21), resulting in an ASN20 ◊ HIS20 substitution.
(TXT)

S3 Dataset Phytochemicals exhibiting strong binding affinities in molecular docking. A list of 298 phytochemicals showing binding affinity scores ≤ −7.5 kcal/mol in molecular docking is provided. PubChem IDs of listed phytochemicals and corresponding source plants are also included.
(XLSX)

S4 Dataset Phytochemicals with optimum physicochemical and drug-likeness properties. Physiochemical and drug-likeness parameters, molecular weight, heavy atoms, aromatic heavy atoms, fraction Csp3, rotatable bonds, H-bond acceptors, H-bond donors, molar refractivity, TPSA, XLOGP3, WLOGP, consensus log P, ESOL log S, log Kp (cm/s), with the Lipinski rule are reported for corresponding phytochemicals.
(XLSX)

S5 Dataset Non-bonded interaction of phytochemicals satisfying ADMET properties. Non-bonded interactions, including hydrogen bond (conventional, hydrogen-carbon, pi-donor), hydrophobic bond (alkyl, pi-alkyl, pi-pi stacked, pi-sulfur, pi-cation, pi-sigma), and van der Waals interaction were analyzed. Moreover, the common interactive residue and interaction number with GM1 or with pentamer formation sites were reported. *ctxB1* and *ctxB7* exhibited similar interaction patterns, and *ctxB3* showed slight distinct interaction profiles.
(XLSX)

S6 Dataset Per-residue energy decomposition of ctxB-phytochemical complexes. Per-residue contributions to the binding energy for all complexes are presented. Residues contribute moderately (≤−0.2 kJ/mol) to favorably (≤−0.5 kJ/mol), and their individual energy components (VdW-van der Waals force, Elec-electrostatic energy contribution, Pol-polar solvation energy, and SASA-nonpolar solvation energy) are presented. A diverse set of amino acid residues was identified as major contributors to binding.
(XLSX)

S7 Dataset Comparison of non-bonded interactions of before and after simulations. Non-bonded interaction (hydrogen, hydrophobic, van der Waals, and others) of complexes between ctxB genotypes (*ctxB3*, *ctxB7*) and five selected phytochemicals are compared before and after molecular dynamic simulations, highlighting changes in interacting residues.
(XLSX)

S1 Fig 2D visualizations of the other four candidate phytochemicals complexed with ctxB. Here, two different types of green circles (dark and extra-light) represent amino acids involved in hydrogen bonds, such as, dark green circle refers to conventional hydrogen bonds, and the extra-light green circle refers to carbon-hydrogen and pi-donor bonds. Textualized forms of amino acid and positions highlighted by light green color represent van der Waals forces. Pink circle represents amino acids involved in hydrophobic bonds, and its different color intensity refers to hydrophobic bond types, such as dark pink refers to pi-pi stacked and pi-pi T-shaped bond, and light pink refers to alkyl and pi-alkyl bond. In addition, the yellowish-brown color circle represents the electrostatic (pi-anion) and other (pi-sulfur) bonds. All complexes with *ctxB1* showed identical interactions as the complexes with *ctxB7*. Slight variations in binding types and positions were observed for complexes with *ctxB3*.
(TIF)

S2 Fig Molecular dynamics simulation results by YASARA for *ctxB3*-*A. indica* (Mol 2) and *ctxB7*-*A. indica* (Mol 2) complexes. (A) RMSD of protein backbone, (B) ligand movement RMSD, (C) ligand conformation RMSD, (D) radius of gyration, (E) protein residue root mean square fluctuation (RMSF), (F) number of hydrogen bonds in *ctxB3*-*A. indica* (Mol 2) complex, and (G) number of hydrogen bonds in *ctxB7*-*A. indica* (Mol 2) complex. In each of the plots, horizontal axis represents simulation time (in ns), and vertical axis represents the measured values of different analyses, except for (E), where horizontal axis represents residue index. The green line indicates *ctxB3*-*A. indica* (Mol 2) complex, and the blue line indicates *ctxB7*-*A. indica* (Mol 2) complex. Ligand movement RMSD for *ctxB7*-*A. indica* (Mol 2) complex fluctuated mainly at 25 ns, suggesting that the ligand may have explored binding pockets other than the initial site. In contrast, the ligand remained in the pocket for *ctxB3*-*A. indica* (Mol 2) complex.
(TIF)

S3 Fig Molecular dynamics simulation results by YASARA for *ctxB3*-*M. alba* (Mol 10) and *ctxB7*-*M. alba* (Mol 10) complexes. (A) RMSD of protein backbone, (B) ligand movement RMSD, (C) ligand conformation RMSD, (D) radius of gyration, (E) protein residue root mean square fluctuation (RMSF), (F) number of hydrogen bonds in *ctxB3*-*M. alba* (Mol 10) complex, and (G) number of hydrogen bonds in *ctxB7*-*M. alba* (Mol 10) complex. In each of the plots, horizontal axis represents simulation time (in ns), and vertical axis represents the measured values of different analyses, except for (E), where horizontal axis represents residue index. The green line indicates *ctxB3*-*M. alba* (Mol 10) complex, and the blue line indicates *ctxB7*-*M. alba* (Mol 10) complex. Ligand movement RMSD for *ctxB3*-*M. alba* (Mol 10) complex shifted at the beginning of the simulation, and after which it remained stable. In contrast, ligand movement RMSD for *ctxB7*-*M. alba* (Mol 10) complex fluctuated notably around 25 ns, 45 ns, 70 ns, suggesting that the ligand may have explored several binding pockets beyond the initial site, with the changes in ligand conformation.
(TIF)

S4 Fig Molecular dynamics simulation results by YASARA for *ctxB3*-*A. bidentata* (Mol 1) and *ctxB7*-*A. bidentata* (Mol 1) complexes. (A) RMSD of protein backbone, (B) ligand movement RMSD, (C) ligand conformation RMSD, (D) radius of gyration, (E) protein residue root mean square fluctuation (RMSF), (F) number of hydrogen bonds in *ctxB3*-*M. bidentata* (Mol 1) complex, and (G) number of hydrogen bonds in *ctxB7*-*M. bidentata* (Mol 1) complex. In each of the plots, horizontal axis represents simulation time (in ns), and vertical axis represents measured values of different analyses, except for (E), where the horizontal axis represents residue index. The green line indicates *ctxB3*-*M. bidentata* (Mol 1) complex, and the blue line indicates *ctxB7*-*M. bidentata* (Mol 1) complex. Ligand movement RMSD for *ctxB3*-*M. bidentata* (Mol 1) complex shifted at the beginning of the simulation, and after which it remained stable. Whereas, ligand movement RMSD for *ctxB7*-*M. bidentata* (Mol 1) complex fluctuated shortly after simulation started and around 10 ns, suggesting that the ligand may have explored multiple binding pockets beyond the initial site.
(TIF)

S5 Fig Molecular dynamics simulation results by YASARA for *ctxB3*-*B. flabellifer* (Mol 2) and *ctxB7*-*B. flabellifer* (Mol 2) complexes. (A) RMSD of protein backbone, (B) ligand movement RMSD, (C) ligand conformation RMSD, (D) radius of gyration, (E) protein residue root mean square fluctuation (RMSF), (F) number of hydrogen bonds in *ctxB3*-*B. flabellifer* (Mol 2) complex, and (G) number of hydrogen bonds in *ctxB7*-*B. flabellifer* (Mol 2) complex. In each of the plots, horizontal axis represents simulation time (in ns), and vertical axis represents measured values of different analyses, except for (E), where horizontal axis represents residue index. The green line indicates *ctxB3*-*B. flabellifer* (Mol 2) complex, and the blue line indicates *ctxB7*-*B. flabellifer* (Mol 2) complex. Ligand movement RMSD for *ctxB3*-*B. flabellifer* (Mol 2) complex gradually increased until 60 ns, and after which it remained stable. Ligand movement RMSD for *ctxB7*-*B. flabellifer* (Mol 2) complex increased gradually until 65 ns, followed by a drift, suggesting that the ligand may have shifted from the initial binding pocket thereafter.
(TIF)

S6 Fig Secondary structure analysis of complexes of *A. indica* (Mol 1) with *ctxB3* (left) and *ctxB7* (right) over the entire 100 ns simulation time, analyzed using YASARA. **A and B** show overall protein secondary structure content (%), and **C and D** represent per-residue secondary structure throughout the simulation, illustrating residue-level structural stability and transitions over time. Blue indicates α-helix, red indicates β-sheet, green indicates coil, cyan indicates turn, yellow indicates 310-helix, and orange indicates π-helix. **E and F** show per-residue ligand contact profiles with *ctxB3* and *ctxB7*, respectively. Here, red indicates hydrogen bonds, green indicates hydrophobic bonds, and blue indicates ionic interactions. Also, mixtures of these three colors can show up if a certain residue is involved in more than one type of contact with the ligand. Overall, the dominant secondary structural elements remain stable throughout the simulation for both complexes.
(TIF)

S7 Fig Secondary structure analysis of complexes of *A. indica* (Mol 2) with *ctxB3* (left) and *ctxB7* (right) over the entire 100 ns simulation time, analyzed using YASARA. **A and B** show overall protein secondary structure content (%), and **C and D** represent per-residue secondary structure throughout the simulation, illustrating residue-level structural stability and transitions over time. Blue indicates α-helix, red indicates β-sheet, green indicates coil, cyan indicates turn, yellow indicates 310-helix, and orange indicates π-helix. **E and F** show per-residue ligand contact profiles with *ctxB3* and *ctxB7*, respectively. Here, red indicates hydrogen bonds, green indicates hydrophobic bonds, and blue indicates ionic interactions. Also, mixtures of these three colors can show up if a certain residue is involved in more than one type of contact with the ligand. Overall, the dominant secondary structural elements remain stable throughout the simulation for both complexes.
(TIF)

S8 Fig Secondary structure analysis of complexes of *M. alba* (Mol 10) with *ctxB3* (left) and *ctxB7* (right) over the entire 100 ns simulation time, analyzed using YASARA. **A and B** show overall protein secondary structure content (%), and **C and D** represent per-residue secondary structure throughout the simulation, illustrating residue-level structural stability and transitions over time. Blue indicates α-helix, red indicates β-sheet, green indicates coil, cyan indicates turn, yellow indicates 310-helix, and orange indicates π-helix. **E and F** show per-residue ligand contact profiles with *ctxB3* and *ctxB7*, respectively. Here, red indicates hydrogen bonds, green indicates hydrophobic bonds, and blue indicates ionic interactions. Also, mixtures of these three colors can show up if a certain residue is involved in more than one type of contact with the ligand. Overall, the dominant secondary structural elements remain stable throughout the simulation for both complexes.
(TIF)

S9 Fig Secondary structure analysis of complexes of *A. bidentata* (Mol 1) with *ctxB3* (left) and *ctxB7* (right) over the entire 100 ns simulation time, analyzed using YASARA. **A and B** show overall protein secondary structure content (%), and **C and D** represent per-residue secondary structure throughout the simulation, illustrating residue-level structural stability and transitions over time. Blue indicates α-helix, red indicates β-sheet, green indicates coil, cyan indicates turn, yellow indicates 310-helix, and orange indicates π-helix. **E and F** show per-residue ligand contact profiles with *ctxB3* and *ctxB7*, respectively. Here, red indicates hydrogen bonds, green indicates hydrophobic bonds, and blue indicates ionic interactions. Also, mixtures of these three colors can show up if a certain residue is involved in more than one type of contact with the ligand. Overall, the dominant secondary structural elements remain stable throughout the simulation for both complexes.
(TIF)

S10 Fig Secondary structure analysis of complexes of *B. flabellifer* (Mol 2) with *ctxB3* (left) and *ctxB7* (right) over the entire 100 ns simulation time, analyzed using YASARA. **A and B** show overall protein secondary structure content (%), and **C and D** represent per-residue secondary structure throughout the simulation, illustrating residue-level structural stability and transitions over time. Blue indicates α-helix, red indicates β-sheet, green indicates coil, cyan indicates turn, yellow indicates 310-helix, and orange indicates π-helix. **E and F** show per-residue ligand contact profiles with *ctxB3* and *ctxB7*, respectively. Here, red indicates hydrogen bonds, green indicates hydrophobic bonds, and blue indicates ionic interactions. Also, mixtures of these three colors can show up if a certain residue is involved in more than one type of contact with the ligand. Overall, the dominant secondary structural elements remain stable throughout the simulation for both complexes.
(TIF)

S11 Fig Molecular dynamics simulation results by GROMACS for *ctxB3*-*A. indica* (Mol 1) and *ctxB7*-*A. indica* (Mol 1) complexes. (A) RMSD of protein backbone, (B) ligand movement RMSD, (C) ligand conformation RMSD, (D) radius of gyration, (E) protein residue root mean square fluctuation (RMSF), (F) number of hydrogen bonds in *ctxB3*-*A. indica* (Mol 2) complex, and (G) number of hydrogen bonds in *ctxB7*-*A. indica* (Mol 1) complex. In each of the plots, horizontal axis represents simulation time (in ns), and vertical axis represents the measured values of different analyses, except for (E), where horizontal axis represents residue index. The green line indicates *ctxB3*-*A. indica* (Mol 1) complex, and the blue line indicates *ctxB7*-*A. indica* (Mol 1) complex. Ligand movement RMSD for *ctxB3*-*A. indica* (Mol 1) complex shifted at the beginning of the simulation, and after which it remained stable. Ligand movement RMSD for *ctxB7*-*A. indica* (Mol 1) complex gradually increased, suggesting that the ligand may have steadily shifted from the initial binding pocket.
(TIF)

S12 Fig Molecular dynamics simulation results by GROMACS for *ctxB3*-*A. indica* (Mol 2) and *ctxB7*-*A. indica* (Mol 2) complexes. (A) RMSD of protein backbone, (B) ligand movement RMSD, (C) ligand conformation RMSD, (D) radius of gyration, (E) protein residue root mean square fluctuation (RMSF), (F) number of hydrogen bonds in *ctxB3*-*A. indica* (Mol 2) complex, and (G) number of hydrogen bonds in *ctxB7*-*A. indica* (Mol 2) complex. In each of the plots, horizontal axis represents simulation time (in ns), and vertical axis represents the measured values of different analyses, except for (E), where horizontal axis represents residue index. The green line indicates *ctxB3*-*A. indica* (Mol 2) complex, and the blue line indicates *ctxB7*-*A. indica* (Mol 2) complex. Ligand movement RMSD for both complexes of *A. indica* (Mol 2) with *ctxB3* and *ctxB7* faced several drifts until 20 ns, and stabilized thereafter.
(TIF)

S13 Fig Molecular dynamics simulation results by GROMACS for *ctxB3*-*M. alba* (Mol 10) and *ctxB7*-*M. alba* (Mol 10) complexes. (A) RMSD of protein backbone, (B) ligand movement RMSD, (C) ligand conformation RMSD, (D) radius of gyration, (E) protein residue root mean square fluctuation (RMSF), (F) number of hydrogen bonds in *ctxB3*-*M. alba* (Mol 10) complex, and (G) number of hydrogen bonds in *ctxB7*-*M. alba* (Mol 10) complex. In each of the plots, horizontal axis represents simulation time (in ns), and vertical axis represents the measured values of different analyses, except for (E), where horizontal axis represents residue index. The green line indicates *ctxB3*-*M. alba* (Mol 10) complex, and the blue line indicates *ctxB7*-*M. alba* (Mol 10) complex.
(TIF)

S14 Fig Molecular dynamics simulation results by GROMACS for *ctxB3*-*A. bidentata* (Mol 1) and *ctxB7*-*A. bidentata* (Mol 1) complexes. (A) RMSD of protein backbone, (B) ligand movement RMSD, (C) ligand conformation RMSD, (D) radius of gyration, (E) protein residue root mean square fluctuation (RMSF), (F) number of hydrogen bonds in *ctxB3*-*M. bidentata* (Mol 1) complex, and (G) number of hydrogen bonds in *ctxB7*-*M. bidentata* (Mol 1) complex. In each of the plots, horizontal axis represents simulation time (in ns), and vertical axis represents measured values of different analyses, except for (E), where the horizontal axis represents residue index. The green line indicates *ctxB3*-*M. bidentata* (Mol 1) complex, and the blue line indicates *ctxB7*-*M. bidentata* (Mol 1) complex. Ligand movement RMSD for both complexes of *M. bidentata* (Mol 1) with *ctxB3* and *ctxB7* faced several drifts until 30 ns, and stabilized thereafter.
(TIF)

S15 Fig Molecular dynamics simulation results by GROMACS for *ctxB3*-*B. flabellifer* (Mol 2) and *ctxB7*-*B. flabellifer* (Mol 2) complexes. (A) RMSD of protein backbone, (B) ligand movement RMSD, (C) ligand conformation RMSD, (D) radius of gyration, (E) protein residue root mean square fluctuation (RMSF), (F) number of hydrogen bonds in *ctxB3*-*B. flabellifer* (Mol 2) complex, and (G) number of hydrogen bonds in *ctxB7*-*B. flabellifer* (Mol 2) complex. In each of the plots, horizontal axis represents simulation time (in ns), and vertical axis represents measured values of different analyses, except for (E), where horizontal axis represents residue index. The green line indicates *ctxB3*-*B. flabellifer* (Mol 2) complex, and the blue line indicates *ctxB7*-*B. flabellifer* (Mol 2) complex. Ligand movement RMSD for both complexes of *B. flabellifer* (Mol 2) with *ctxB3* and *ctxB7* faced several drifts until 12 ns, and stabilized thereafter.
(TIF)

S16 Fig MM-PBSA binding free energies (kJ/mol) of all 10 ctxB-phytochemical complexes by YASARA and GROMACS. The left panel contains the results from YASARA software packages, and the right panel contains the results from GROMACS software packages. In each of the plots, the horizontal axis represents simulation time (in ns), and vertical axis represents values of the energy measurements (kJ/mol), green line indicates *ctxB3*-phytochemical complexes, and the blue line indicates *ctxB7*-phytochemical complexes. Recoded names of the phytochemicals corresponding to the complexes are textualized within the figures. Higher positive binding energies indicate favorable binding in YASARA, and more negative binding energies indicate favorable binding in GROMACS. Both software packages showed similar trends in binding energy, consistently indicating favorable interactions.
(TIF)

S17 Fig Per residue binding free energy decomposition of *ctxB-*phytochemical complexes. **Panel A** shows *Azadirachta indica* (Mol 2), and **Panel B** shows *Azadirachta indica* (Mol 1). In each panel, the horizontal axis represents ctxB variants (*ctxB3*, *ctxB7*), and the vertical axis represents the amino acid residues with their positions. Orange color indicates negative (favorable) values, purple indicates positive (unfavorable) values, and white denotes near-zero contributions. A diverse set of top energy contributed amino acids was observed across different ctxB variants.
(TIF)

S18 Fig Per residue binding free energy decomposition for all *ctxB-*phytochemical complexes. The horizontal axis represents the names of complexes, and the vertical axis represents the amino acid residues with their positions. Orange color indicates negative (favorable) values, purple indicates positive (unfavorable) values, and white denotes near-zero contributions. A total of 70 amino acids out of 124 were assembled in this analysis, collectively accounting for ~80% of the total binding energy of each complex, with individual residues contributing favorably (≤−0.5 kJ/mol) to the total binding energy.
(TIF)
