# Supplementary material for: In-silico identification of anti-cholera phytochemicals from Indian medicinal plants
Source: PLoS One. 2026 Feb 2;21(2):e0342058. doi: 10.1371/journal.pone.0342058 (PMC12863543; doi:10.1371/journal.pone.0342058)
Supplement: S1 File — Detailed descriptions of system preparation, simulation parameters, equilibration, production runs, and trajectory analyses (e.g., RMSD, RMSF, Radius of gyration, number of hydrogen bonds, MM-PBSA binding energy) were performed using GROMACS. (DOCX) [file pone.0342058.s001.docx]

**Supplementary methods for GROMACS simulations.**

Molecular dynamics simulations were performed using GROMACS [1]. Protein topology was generated using the CHARMM36 force field [2] in collaboration with the CHARMM-modified three-point transferable intermolecular potential (TIP3P) water model [3], and ligand topology parameters were derived from the CGenFF server. The protein-ligand complex was then assembled, and the topology file (topol.top) was updated accordingly. A dodecahedron simulation box was constructed with a 1 nm solvent buffer surrounding the complex and solvated with water. Each system was then neutralized by adding Na^+^ and Cl^-^ counter-ions. Long-range interactions were treated using the Particle-Mesh Ewald (PME) method [4], and molecular dynamics simulations were performed using the leap-frog integrator [5]. Position restraints were applied to the non-hydrogen atoms of the ligand with a force constant of 1000 kJmol^-1^nm^-2^ applied independently along the x, y, and z directions. Energy minimization was performed using the steepest descent algorithm under periodic boundary conditions (PBC) to remove unfavorable contacts. System equilibration was carried out in two stages under NVT and NPT ensembles. During NVT equilibration, the temperature was maintained at 300K for 1000ps with a 2fs time step using the velocity-rescaling (V-rescale) thermostat. This was followed by NPT equilibration to stabilize pressure at 1 bar using the Berendsen thermostat with isotropic coupling. The equilibrated coordinates were used to perform production MD simulations for 100ns (50000000 steps with a 2-fs time step) under the NPT ensemble using Parrinello-Rahman barostat to maintain correct pressure fluctuations. Trajectories were centered and wrapped before further analysis. To reduce data size, the production trajectory was subsampled every 200 ps while retaining the full system for subsequent analyses. The Root mean square deviation of the backbone (RMSDBb), ligand movement RMSD, ligand conformation RMSD, radius of gyration (Rg), Root mean square fluctuations (RMSF), and number of hydrogen bonds were analyzed from the subsampled trajectory. Binding free energies were calculated using the MM-Poisson-Boltzmann surface area (MM-PBSA) method. The electrostatic energy, van der Waals energy, and polar solvation energy were calculated using the Adaptive Poisson-Boltzmann Solver (APBS). Solvent-accessible surface area (SASA) was used to approximate the non-polar energy contributions. The dielectric constant of 2 was assigned to the solute, and 80 to the solvent, with a vacuum dielectric constant of 1 used as a reference. The overall binding free energy was calculated as:

∆G_bind_=G_complex_-(G_receptor_+G_ligand_)

For per-residue energy decomposition, the binding free energy (∆G_bind_) is expressed as:

∆G_bind_=∆E_mm_+∆G_solv_-T∆S

where ∆E_mm_ is the gas-phase interaction energy of the complex, including van der Waals (∆E_vdw_), electrostatic (∆E_elec_) interactions, and the internal energy variations (∆E_intra_). ∆G_solv_ is the solvation free energy difference, comprising electrostatic solvation free energy (∆G_PB_) and nonpolar contributions (∆G_SA_). -T∆S represents the contributions of the entropy of the solute molecules, but was not considered in the present work.

**Supplementary References**

1. Abraham MJ, Murtola T, Schulz R, Páll S, Smith JC, Hess B, et al. GROMACS: High performance molecular simulations through multi-level parallelism from laptops to supercomputers. SoftwareX. 2015;1–2: 19–25. doi:10.1016/j.softx.2015.06.001

2. Huang J, MacKerell AD. CHARMM36 all-atom additive protein force field: Validation based on comparison to NMR data. J Comput Chem. 2013;34: 2135–2145. doi:10.1002/jcc.23354

3. Price DJ, Brooks CL. A modified TIP3P water potential for simulation with Ewald summation. J Chem Phys. 2004;121: 10096–10103. doi:10.1063/1.1808117

4. Darden T, York D, Pedersen L. Particle mesh Ewald: An *N* ⋅log( *N* ) method for Ewald sums in large systems. J Chem Phys. 1993;98: 10089–10092. doi:10.1063/1.464397

5. Van Gunsteren WF, Berendsen HJC. A Leap-frog Algorithm for Stochastic Dynamics. Mol Simul. 1988;1: 173–185. doi:10.1080/08927028808080941
