# Supplementary material for: In-silico identification of anti-cholera phytochemicals from Indian medicinal plants
Source: PLoS One. 2026 Feb 2;21(2):e0342058. doi: 10.1371/journal.pone.0342058 (PMC12863543; doi:10.1371/journal.pone.0342058)
Supplement: S1 Table — Model quality metrics (QSQE, GMQE), and template information for ctxB alleles are provided. (DOCX) [file pone.0342058.s002.docx]

**S1 Table. Structural prediction details of ctxB toxin alleles using SWISS-MODEL**

| Toxin  Allele | Template | Template ID | Coverage | Seq  Identity | QSQE | GMQE |
| --- | --- | --- | --- | --- | --- | --- |
| *ctxB1* | gene: ctxB,  organism: *Vibrio cholerae*  (Experimental) | PDB ID- 5lzh  Method- X-ray, 1.13 Å | 0.83 | 100% | 0.81 | 0.69 |
| *ctxB7* | gene: ctxB,  organism: *Vibrio cholerae*  (Experimental) | PDB ID- 5lzh  Method- X-ray, 1.13 Å | 0.83 | 100% | 0.81 | 0.69 |
| *ctxB3* | gene: ctxB,  organism: *Vibrio cholerae*  (Experimental) | PDB ID- 5elf  Method- X-ray, 1.55 Å | 0.83 | 100% | 0.77 | 0.70 |

Here, GMQE (Global Model Quality Estimate) refers to a quality measure of the overall predicted protein model, with values closer to 1 indicating better quality, ranging from 0 to 1. QSQE (Quaternary Structure Quality Estimate) refers to the accuracy of interchain contacts in a multi-chain protein complex, with a score above 0.7 considered reliable.
