# Supplementary material for: In-silico identification of anti-cholera phytochemicals from Indian medicinal plants
Source: PLoS One. 2026 Feb 2;21(2):e0342058. doi: 10.1371/journal.pone.0342058 (PMC12863543; doi:10.1371/journal.pone.0342058)
Supplement: S2 Table — Distribution of phytochemicals from individual plant sources across successive screening steps, including molecular docking, drug-likeness, and pharmacokinetic filtering. (DOCX) [file pone.0342058.s003.docx]

**S2 Table. Plant-specific phytochemicals at different screening steps**

| Source Plants | Locally known name (Bangla) | Total no. of selected  phytochemicals | No. of phytochemicals  with the binding affinity (≤ -7.5) kcal/mol | No. of phytochemicals after that satisfied ADME properties |
| --- | --- | --- | --- | --- |
| *Abrus precatorius* | Kunch | 117 |  |  |
| *Acalypha fruticosa* | N/A | 7 | 1 |  |
| *Acalypha indica* | Muktajhuri | 82 | 1 |  |
| *Aconitum ferox* | Vujnag | 8 |  |  |
| *Achyranthes bidentata* | Datiwan | 128 | 7 | 2 |
| *Adenanthera pavonina* | Ranjana | 56 | 2 | 1 |
| *Aegle marmelos* | Indian bael | 151 |  |  |
| *Aerva lanata* | Bhadram | 7 |  |  |
| *Alangium salviifolium* | Ankor kanta | 176 | 3 | 1 |
| *Allium cepa* | Piaj (Onion) | 355 | 6 |  |
| *Allium wallichii* | Jimbur | 8 |  |  |
| *Alstonia scholaris* | Chhatim | 141 | 3 | 1 |
| *Amaranthus spinosus* | Notey shak | 27 |  |  |
| *Andrographis paniculata* | Kalomegh | 211 | 1 | 1 |
| *Areca catechu* | Supari (betel nut) | 62 | 4 | 1 |
| *Azadirachta indica* | Neem | 366 | 7 | 2 |
| *Barringtonia acutangula* | Hijal | 25 |  |  |
| *Bauhinia vahlii* | Chehur lata | 5 |  |  |
| *Berberis aristata* | Dharhaldi | 14 | 1 | 1 |
| *Blumea balsamifera* | Chapor | 89 | 3 |  |
| *Blumea lacera* | Tamrachura | 19 |  |  |
| *Bombax ceiba* | Shimul | 165 | 3 |  |
| *Borassus flabellifer* | Tal (palm) | 10 | 2 | 2 |
| *Calotropis gigantea* | Akanda | 38 | 8 | 2 |
| *Capsicum annuum* | Lanka (long peeper) | 706 | 16 | 2 |
| *Centella asiatica* | Thankuni | 152 | 5 | 1 |
| *Cinnamomum verum* | Daruchini | 99 |  |  |
| *Citrus aurantium* | Bitter orange | 183 | 24 | 1 |
| *Citrus limon* | Lebu (lemon) | 67 | 3 |  |
| *Citrus maxima* | Batabi lebu (pomelo) | 230 | 6 | 3 |
| *Cocos nucifera* | Narikel (Cocunut) | 47 |  |  |
| *Cucumis sativus* | Shasa (Cucumber) | 90 | 3 |  |
| *Curcuma longa* | Halud (Turmeric) | 389 | 4 |  |
| *Cymbopogon citratus* | Lemon grass | 42 |  |  |
| *Diospyros malabarica* | Deshi gab | 1 |  |  |
| *Dillenia indica* | Chalta | 50 | 5 | 1 |
| *Emblica officinalis* | Amloki | 165 | 17 |  |
| *Hordeum vulgare* | Barley | 88 | 1 |  |
| *Jatropha curcas* | Jamal Gota (Vagh barenda) | 28 | 4 |  |
| *Magnolia champaca* | Champa | 40 |  |  |
| *Mangifera indica* | Aam (Mango) | 582 | 7 |  |
| *Mentha spicata* | Pudina (spearmint) | 88 |  |  |
| *Moringa oleifera* | Sajina | 87 | 1 | 1 |
| *Morus alba* | Tut (Mulberry) | 524 | 101 | 11 |
| *Murraya paniculata* | Jui (jasmine) | 322 | 11 | 4 |
| *Musa paradisiaca* | Kola (Banana) | 49 | 2 |  |
| *Nelumbo nucifera* | Padma (lotus) | 186 | 2 |  |
| *Ocimum tenuiflorum* | Tulsi (holy basil) | 147 | 4 |  |
| *Plumeria rubra* | Lal Kathgolap | 57 |  |  |
| *Psidium guajava* | Peyara (Guava) | 166 | 15 | 2 |
| *Sesamum indicum* | Til (flick) | 112 | 8 | 1 |
| *Solanum melongena* | Begun (Eggplant) | 14 |  |  |
| *Vachellia farnesiana* | N/A | 5 |  |  |
| *Vachellia nilotica* | Babla | 31 | 5 |  |
| *Zingiber officinale* | Ada (ginger) | 593 | 2 |  |
| **Total** |  | **7607** | **298** | **41** |

Here, the initially selected total number of phytochemicals from 55 source plants, the local names of source plants, the number of phytochemicals with affinity score ≤ -7.5 kcal/mol from respective plants, and the number of phytochemicals that satisfied drug-likeness properties from 20 different plants were presented.
