# Supplementary material for: In-silico identification of anti-cholera phytochemicals from Indian medicinal plants
Source: PLoS One. 2026 Feb 2;21(2):e0342058. doi: 10.1371/journal.pone.0342058 (PMC12863543; doi:10.1371/journal.pone.0342058)
Supplement: S3 Table — Original phytochemical names, their PubChem ID, and corresponding recoded names are listed. (DOCX) [file pone.0342058.s004.docx]

**S3 Table.**  **Phytochemicals with recoded names**

| Sl. No. | PubChem ID | Source Plant | Recoded names of phytochemicals | **Binding Affinity Score  (kcal/mol)** | | |
| --- | --- | --- | --- | --- | --- | --- |
|  |  |  |  | *ctxB7* | *ctxB1* | *ctxB3* |
| 1 | 196583 | *Morus alba* | *M. alba* (Mol 1) | -8.4 | -8.6 | -8.6 |
| 2 | 118701648 | *Morus alba* | *M. alba* (Mol 2) | -8.7 | -8.2 | -8.6 |
| 3 | 9959532 | *Morus alba* | *M. alba* (Mol 3) | -8.1 | -8.2 | -8.6 |
| 4 | 102339032 | *Morus alba* | *M. alba* (Mol 4) | -8.4 | -8.4 | -7.8 |
| 5 | 441780 | *Morus alba* | *M. alba* (Mol 5) | -8 | -8.2 | -8.3 |
| 6 | 44567218 | *Morus alba* | *M. alba* (Mol 6) | -8.3 | -7.9 | -8.1 |
| 7 | 102384790 | *Morus alba* | *M. alba* (Mol 7) | -8.6 | -8.1 | -7.6 |
| 8 | 5481972 | *Morus alba* | *M. alba* (Mol 8) | -8 | -8 | -7.9 |
| 9 | 162957955 | *Morus alba* | *M. alba* (Mol 9) | -7.6 | -8 | -7.7 |
| 10 | 145955804 | *Morus alba* | *M. alba* (Mol 10) | -7.8 | -7.8 | -7.5 |
| 11 | 5481973 | *Morus alba* | *M. alba* (Mol 11) | -7.5 | -7.8 | -7.6 |
| 12 | 126009 | *Murraya paniculata* | *M. paniculata* (Mol 1) | -9 | -9 | -9.1 |
| 13 | 5315364 | *Murraya paniculata* | *M. paniculata* (Mol 2) | -8.6 | -8.6 | -8.5 |
| 14 | 442977 | *Murraya paniculata* | *M. paniculata* (Mol 3) | -8.4 | -8.4 | -7.5 |
| 15 | 5315354 | *Murraya paniculata* | *M. paniculata* (Mol 4) | -7.6 | -7.6 | -8 |
| 16 | 119041 | *Citrus maxima* | *C. maxima* (Mol 1) | -7.9 | -7.9 | -7.8 |
| 17 | 20055680 | *Citrus maxima* | *C. maxima* (Mol 2) | -7.9 | -7.8 | -7.8 |
| 18 | 6710776 | *Citrus maxima* | *C. maxima* (Mol 3) | -7.7 | -7.6 | -7.8 |
| 19 | 99474 | *Achyranthes bidentata* | *A. bidentata* (Mol 1) | -7.8 | -7.8 | -7.9 |
| 20 | 91453 | *Achyranthes bidentata* | *A. bidentata* (Mol 2) | -8 | -7.7 | -7.5 |
| 21 | 442153 | *Azadirachta indica* | *A. indica* (Mol 1) | -8 | -8 | -7.5 |
| 22 | 52951893 | *Azadirachta indica* | *A. indica* (Mol 2) | -7.5 | -7.5 | -7.5 |
| 23 | 11827970 | *Borassus flabellifer* | *B. flabellifer* (Mol 1) | -8 | -8 | -8.1 |
| 24 | 441900 | *Borassus flabellifer* | *B. flabellifer* (Mol 2) | -7.8 | -7.8 | -7.9 |
| 25 | 65727 | *Capsicum annuum* | *C. annuum* (Mol 1) | -7.8 | -7.8 | -7.8 |
| 26 | 11050123 | *Capsicum annuum* | *C. annuum* (Mol 2) | -7.7 | -7.6 | -7.5 |
| 27 | 441874 | *Calotropis gigantea* | *C. gigantea* (Mol 1) | -8.1 | -8.2 | -7.6 |
| 28 | 441844 | *Calotropis gigantea* | *C. gigantea* (Mol 2) | -8 | -8 | -7.5 |
| 29 | 155517916 | *Psidium guajava* | *P. guajava* (Mol 1) | -7.9 | -7.8 | -7.6 |
| 30 | 162853621 | *Psidium guajava* | *P. guajava* (Mol 2) | -7.7 | -7.6 | -7.7 |
| 31 | 5281644 | *Areca catechu* | *A. catechu* (Mol 1) | -7.9 | -7.9 | -8.2 |
| 32 | 637463 | *Andrographis paniculata* | *A. paniculata* (Mol 1) | -7.8 | -7.8 | -7.6 |
| 33 | 138113455 | *Adenanthera pavonina* | *A. pavonina* (Mol 1) | -7.9 | -7.8 | -8.3 |
| 34 | 165327 | *Alangium salviifolium* | *A. salviifolium* (Mol 1) | -8.4 | -8.6 | -7.7 |
| 35 | 139041048 | *Alstonia scholaris* | *A. scholaris* (Mol 1) | -7.6 | -7.6 | -7.5 |
| 36 | 362574 | *Berberis aristata* | *B. aristata* (Mol 1) | -7.9 | -7.9 | -7.9 |
| 37 | 10719817 | *Centella asiatica* | *C. asiatica* (Mol 1) | -8 | -8 | -7.8 |
| 38 | 90656863 | *Citrus aurantium* | *C. aurantium* (Mol 1) | -8.7 | -8.1 | -8.3 |
| 39 | 12310964 | *Dillenia indica* | *D. Indica* (Mol 1) | -7.5 | -7.5 | -7.6 |
| 40 | 92966492 | *Moringa oleifera* | *M. oleifera* (Mol 1) | -7.7 | -7.6 | -7.9 |
| 41 | 67805702 | *Sesamum indicum* | *S. indicum* (Mol 1) | -8 | -7.7 | -7.6 |

Here, phytochemicals from the same source plant were recoded with a prefix of the respective plant name. The PubChem ID and docking affinity scores of corresponding phytochemicals against three genotypes of ctxB were also presented.
