# Supplementary material for: In-silico identification of anti-cholera phytochemicals from Indian medicinal plants
Source: PLoS One. 2026 Feb 2;21(2):e0342058. doi: 10.1371/journal.pone.0342058 (PMC12863543; doi:10.1371/journal.pone.0342058)
Supplement: S4 Table — Key amino acid residues involved in ligand binding and toxin functionality are reported. (DOCX) [file pone.0342058.s005.docx]

**S4 Table. The active residues of ctxB**

| Interacting residues of GM1 and ctxB  (PDB ID-3CHB) | Interacting residues of LewisX and ctxB  (PDB ID-6hjd) | Interacting residues of pentamer formation of ctxB itself  (PDB ID-5elb, 3CHB, 1eei) |
| --- | --- | --- |
| ASN111, GLN77, GLN82, GLU32, GLU72, HIS34, LYS112, TYR33, ILE79, HIS78, TRP109 | HIS39, HIS115, ASN65, GLY66, ALA67, PHE69, THR68, PRO114, GLN27, THR113 | ALA53, LEU52, ALA85, LEU98, ALA101, LEU29, ALA118, LYS84, ARG56, MET122, ARG88, MET89, ARG94, SER121, ASP91, SER81, GLN82, THR22, GLU32, THR49, GLU50, THR92, GLU57, THR113, GLU87, TRP109, GLY54, TYR33, ILE86, TYR48, ILE95, TYR97, ILE120 |

Here, all residues combinedly indicate the active amino acids of ctxB.
